# β-V_2_O_5_ as Magnesium Intercalation Cathode

**DOI:** 10.1021/acsaem.2c02371

**Published:** 2022-10-03

**Authors:** Rafael Trócoli, Prakash Parajuli, Carlos Frontera, Ashley P. Black, Grant C. B. Alexander, Indrani Roy, M. Elena Arroyo-de Dompablo, Robert F. Klie, Jordi Cabana, M. Rosa Palacín

**Affiliations:** † Instituto de Ciencia de Materiales de Barcelona (ICMAB-CSIC), Campus de la UAB, 08193 Bellaterra, Catalonia, Spain; ‡ Departamento de Química Inorgánica e Ingeniería Química, Instituto Universitario de Nanoquímica (IUNAN), Facultad de Ciencias, Universidad de Córdoba, Campus de Rabanales, Córdoba 14071, Spain; § Department of Chemistry, University of Illinois at Chicago, Chicago, Illinois 60607, United States; ∥ Department of Physics, University of Illinois at Chicago, Chicago, Illinois 60607, United States; ⊥ Departamento de Química Inorgánica, Universidad Complutense de Madrid, Madrid 28040, Spain; # Joint Center for Energy Storage Research, Argonne National Laboratory, Argonne, Illinois 60439, United States

**Keywords:** magnesium batteries, vanadium oxide, magnesium intercalation, operando XRD, β-V_2_O_5_

## Abstract

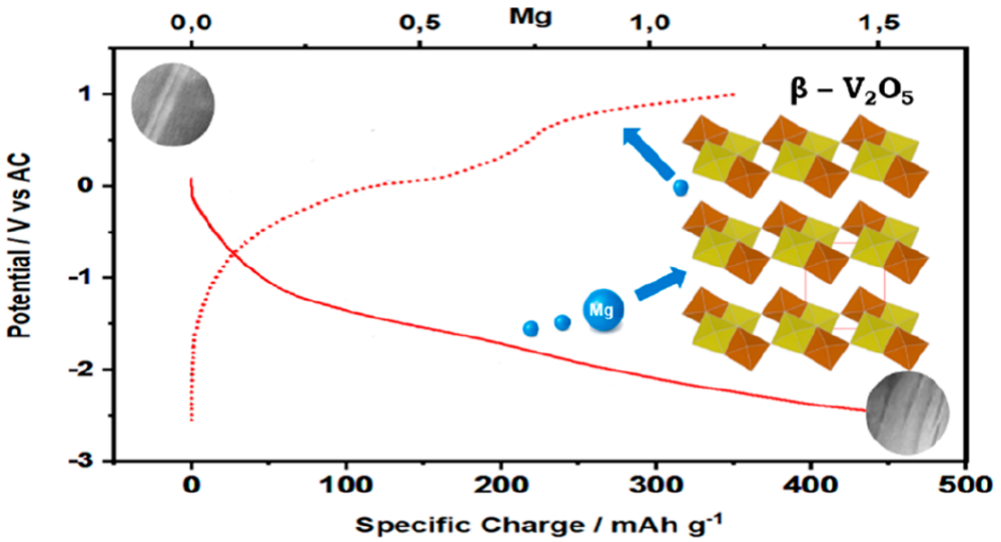

Magnesium batteries have attracted great attention as an alternative to Li-ion batteries but still suffer from limited choice of positive electrode materials. V_2_O_5_ exhibits high theoretical capacities, but previous studies have been mostly limited to α-V_2_O_5_. Herein, we report on the β-V_2_O_5_ polymorph as a Mg intercalation electrode. The structural changes associated with the Mg^2+^ (de-) intercalation were analyzed by a combination of several characterization techniques: *in situ* high resolution X-ray diffraction, scanning transmission electron microscopy, electron energy-loss spectroscopy, and X-ray absorption spectroscopy. The reversible capacity reached 361 mAh g^–1^, the highest value found at room temperature for V_2_O_5_ polymorphs.

Notwithstanding the ubiquitous presence of lithium-ion batteries (LIBs) in portable applications, research in alternative technologies based on abundant elements which could provide higher energy density is currently expanding. Among them, magnesium metal batteries have emerged as one of the most promising candidates. Magnesium exhibits high volumetric capacity (3833 mAh cm^–3^), low reduction potential (−2.4 V vs SHE), and a lower tendency to form dendrites than Li. However, practical deployment of these batteries is plagued with diverse hurdles such as the lack of stable electrolytes featuring wide electrochemical windows, suitable kinetics at the metal magnesium electrode and the absence of positive electrodes operating at high potential.^1−5^ Moreover, sluggish solid-state diffusion of Mg^2+^, caused by the strong Coulombic interactions between Mg^2+^ and host material (the charge to radius ratio is doubled for Mg^2+^ when compared to Li^+^), is an additional bottleneck to overcome. Vanadium pentoxide (V_2_O_5_) has recently attracted large attention due to its high theoretical capacity associated with the rich redox chemistry of Vanadium. In contrast with the large number of studies devoted to α-V_2_O_5_^6−11^ (its structure is showed in Figure 1a), other polymorphs have deserved less attention, probably due to their more complex synthesis route. The ζ-V_2_O_5_ metastable polymorph has been shown to exhibit an average operating voltage of 1.65 V versus Mg/Mg^2+^, yet modest stable capacities (90 mAh g^–1^)^12^ greatly improved (140 mAh g ^–1^) through reducing the particle size down to ca. 100 nm.^13^ It is important to note that the electrochemical studies displaying the most conclusive evidence of reversible Mg^2+^ intercalation were conducted at high temperature (*T* = 50–110 °C) to overcome significant limitations of efficiency.^14^

**Figure 1:**
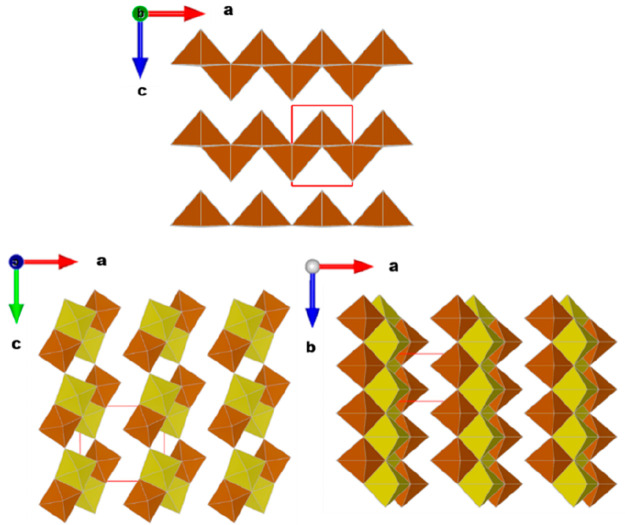
Schematic representation of the α-V_2_O_5_ (a) and β-V_2_O_5_ (b) structures. α-V_2_O_5_ data are taken from The Materials Project (mp-25279). β-V_2_O_5_ structure is based on *a* = 7.1140(2) Å, *b* = 3.5718(1) Å, *c* = 6.2846(2) Å, α = 90°, β = 90.069(3)°, γ = 90°. Yellow and brown polyhedra represent the highly distorted VO_6_ octahedra at positions V1 and V2, respectively. Crystal structure data taken from P. Filonenko et al.^18^

The high pressure β-V_2_O_5_ polymorph has been shown to insert up to 2.0 Li^+^ or 1.0 Na^+^ per formula unit but to the best of our knowledge has not been previously investigated in magnesium cells.^15−17^ Its crystal structure (monoclinic, *P*2_1_/*m*, Figure 1c) can be described as built up of infinite chains of quadruple units of edge-sharing VO_6_ octahedra along the short *b* axis and mutually linked by corner-sharing between two octahedra along *c*, thus forming two-dimensional layers of composition V_4_O_10_ in planes parallel to (100).^15,18^ Herein we present proof of the feasibility of magnesium insertion and deinsertion in β-V_2_O_5_, characterized using different complementary techniques.

To the best of our knowledge, β-V_2_O_5_ showed the highest deinsertion capacity of a magnesium positive electrode at room temperature, which might even be improved via strategies such as particle nanoengineering and cell chemistry optimization.

β-V_2_O_5_ was synthesized at high pressure (see experimental details) following the protocol described by Arroyo-de Dompablo *et al*.^16,19,20^ and is a black-reddish powder constituted by needle-like particles with ≈70 nm width by 1–2 μm length and also smaller platelets a few hundred nanometers wide (Figure 2a). Its XRD pattern could be successfully refined considering the crystal structure proposed by V. P. Filonenko et al.^18^ (Figure 2b).

**Figure 2:**
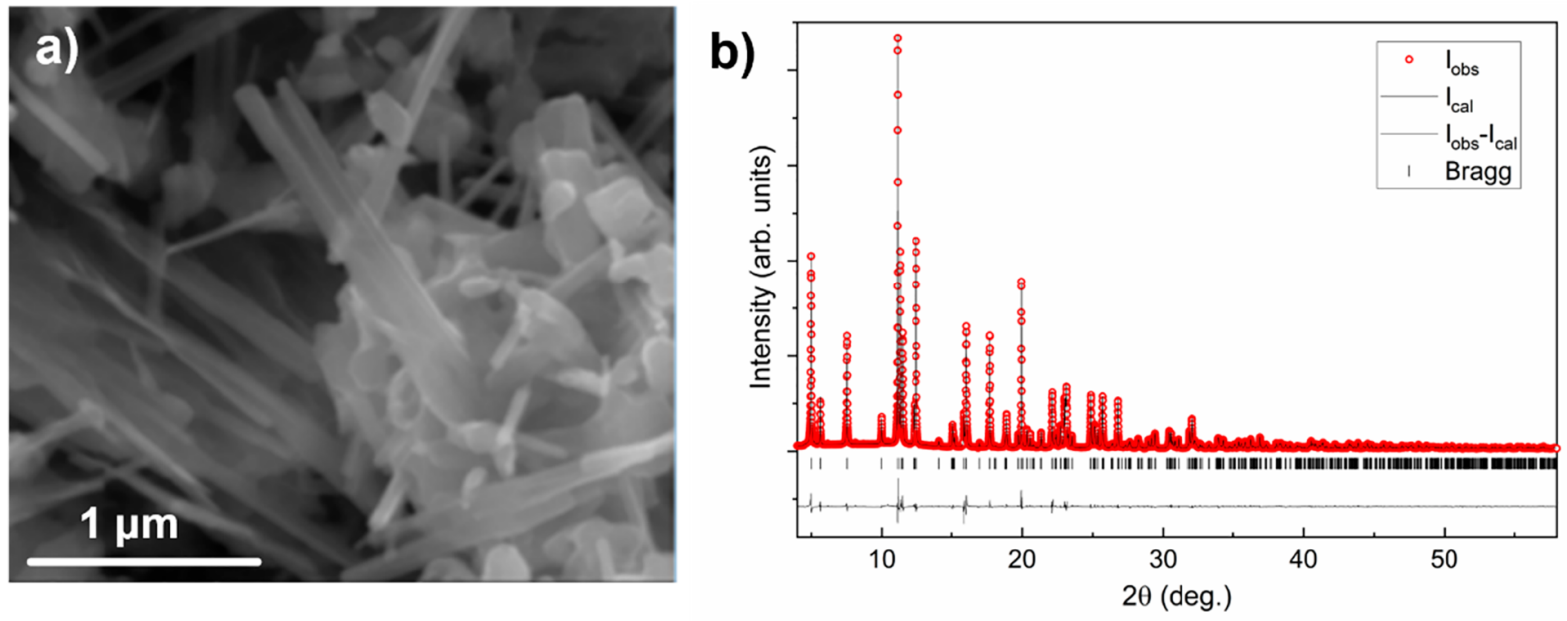
Representative SEM images (a) and synchrotron X-ray diffraction (SXRD) pattern and corresponding Rietveld refinement for as prepared β-V_2_O_5_ (b). λ = 0.6192 Å.

Electrochemical experiments aiming at inserting magnesium in β-V_2_O_5_ were carried out using dry 0.1 M Mg(ClO_4_)_2_ in acetonitrile as electrolyte, with <20 ppm of water, as deduced from Karl Fischer titration. Figure 3a shows the results of cyclic voltammetry, CV (selected cycles). During the first reduction, a sharp peak at −2.29 V vs Ag, potentially associated with Mg insertion, was observed. On the contrary, its oxidation counterpart at around 0.6 V vs Ag, tentatively associated with Mg deinsertion, is much broader and exhibits lower amplitude. The CV profile evolves upon cycling with gradual widening of the reduction peak and sharpening of the oxidation peak, with the latter also shifting its position to lower potential (≈0.41 V vs Ag). Similar tendencies were observed during the studies at a constant current equivalent to C/25 (Figure 3b). In the first cycle, a sloping profile was observed for β-V_2_O_5_ oxidation and reduction with large polarization (≈2 V). Figure 3c shows the evolution of the specific capacity upon cycling, which increased from close to 157 mAh g^–1^ in the first cycle up to 361 mAh g^–1^ in the 14th cycle, concomitant to a decrease in polarization (Figure 3b). The difference observed between reduction and oxidation is associated with the trapping of Mg in the β-V_2_O_5_ structure and likely also to side reactions involving electrolyte decomposition.

**Figure 3:**
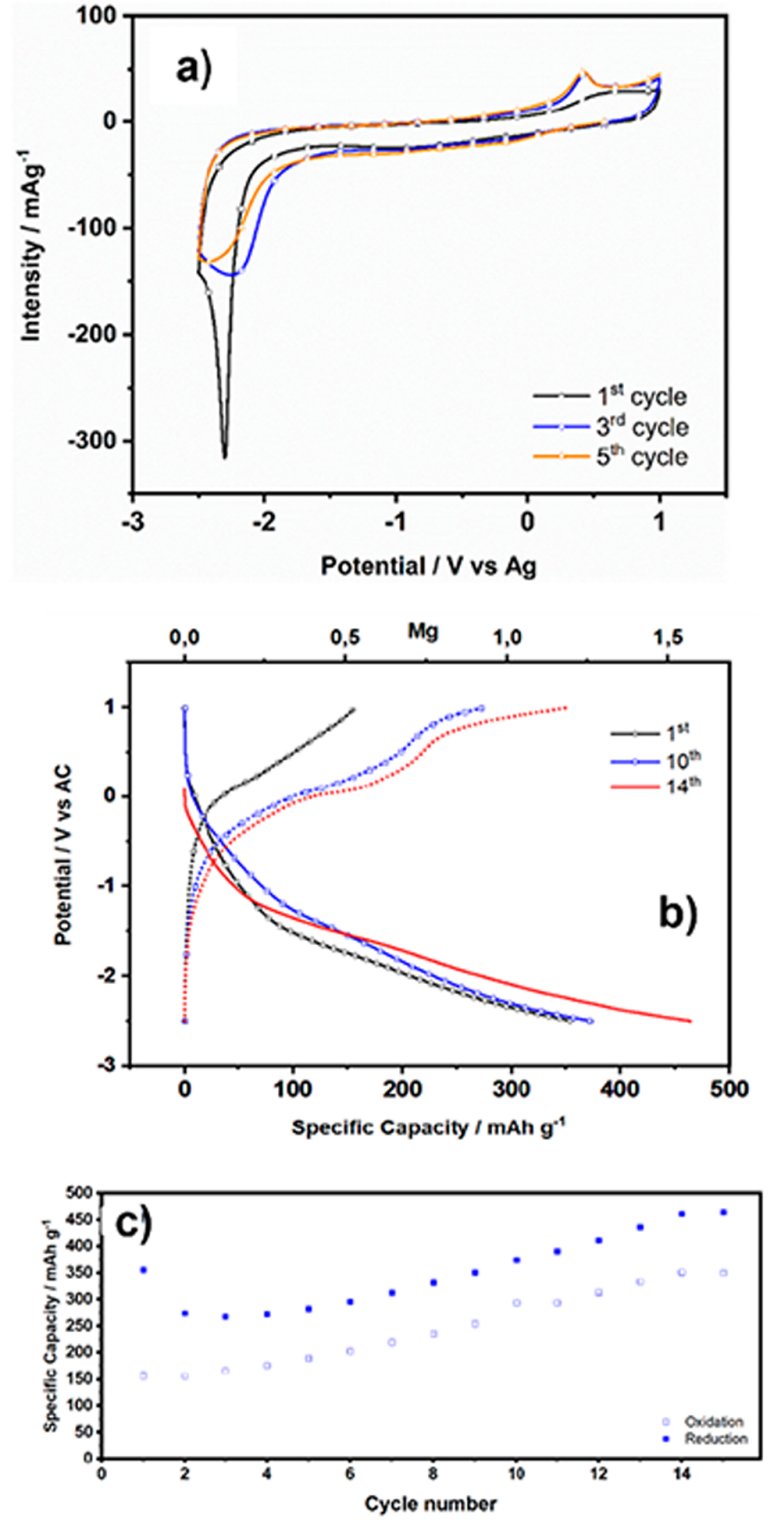
Selected cyclic voltammetry (a), reduction/oxidation cycles in galvanostatic mode (b), and specific capacity evolution upon cycling (c) for β-V_2_O_5_ using 0.1 Mg(ClO_4_)_2_ in acetonitrile as electrolyte. AC = activated carbon.

Representative STEM images corresponding to β-V_2_O_5_ as prepared, after reduction, and after reoxidation in magnesium cells are depicted in Figure 4. The comparison reveals that the reduction process induced considerable delamination of the particles, which remained after further oxidation (Figure 4c). Homogeneous distribution of both V and Mg was observed in the reduced and reoxidized samples (a fraction of Mg was not deinserted) by elemental mapping (Figure 4d–f). This is in agreement with Mg insertion into crystal structure rather than a conversion reaction taking place, as the latter would introduce significant inhomogeneity because of phase separation.^21^

**Figure 4:**
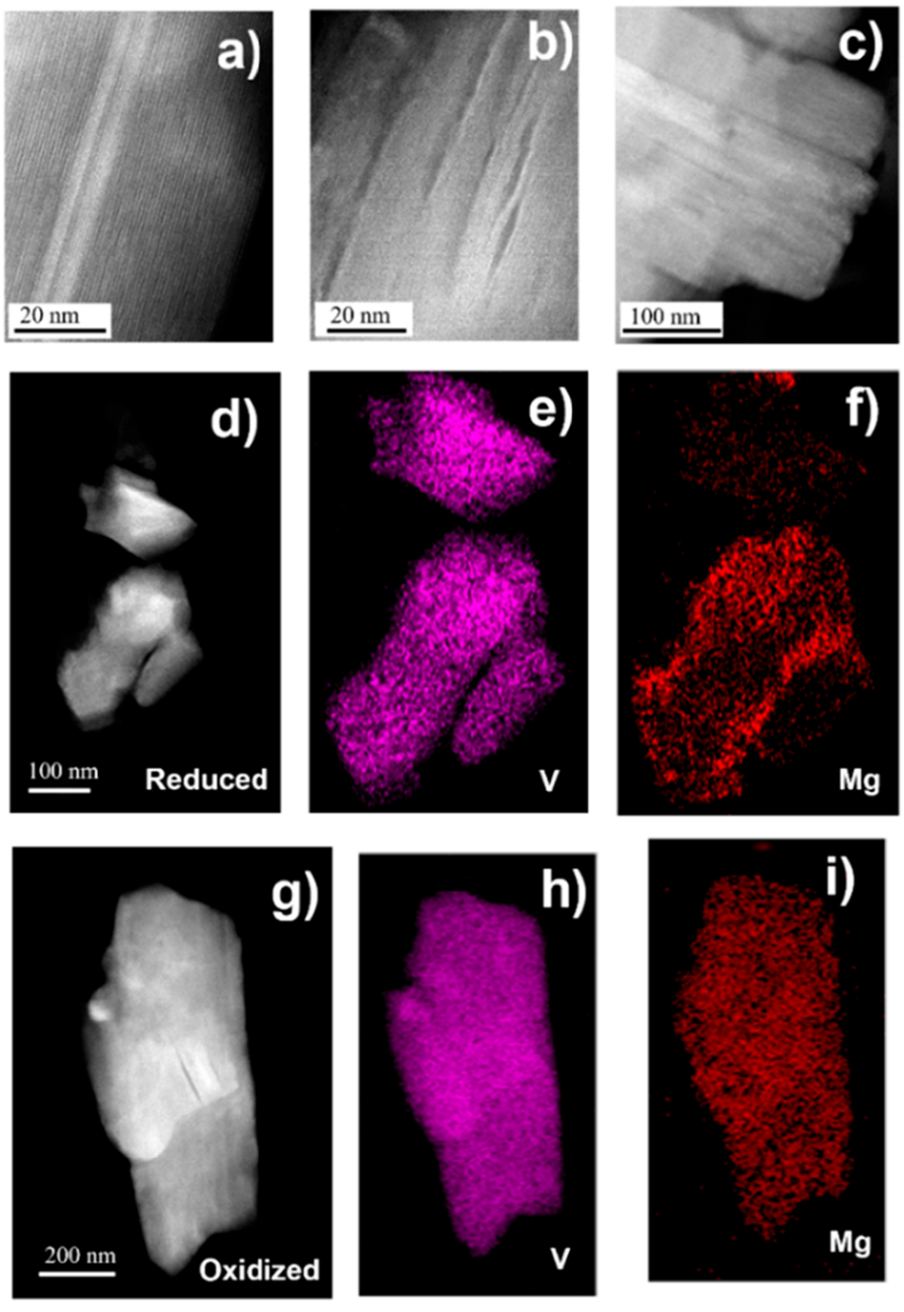
High angle annular dark-field images of pristine (a), reduced (b, d), and reoxidized (c, g) samples which enabled to detect morphological and compositional changes. Elemental mapping of reduced (e, f) and reoxidized (h, i) particles for V and Mg are also shown.

The striking decrease in size of the crystal domains could contribute to the decrease in voltage hysteresis observed upon cycling (Figure 3b), a behavior that was also observed in α-V_2_O_5_.^7^

Average compositions of β-Mg_0.62_V_2_O_5_ and β-Mg_0.20_V_2_O_5_ for reduced and reoxidized samples were deduced from EDS spectra, which agree with the electrochemical capacities achieved, β-Mg_0.78_V_2_O_5_ (reduced) and β-Mg_0.15_V_2_O_5_ (reoxidized). The EEL spectra from the pristine and cycled materials are shown in Figure 5. The energy scales are calibrated with respect to the zero-loss peak. The background is removed by a power law fitting, and the intensity is normalized to the V L_3_-edge peak intensity. Figure 5 shows that the energy of the V L_2,3_*-*edge decreases by approximately 1 eV from pristine to reduced states, accompanied by a shift to higher energy and modulation of the fine structure of the O K-edge between 530 and 535 eV, indicating a change in the states involving O 2p-V 3d hybridization. Overall, the changes at both edges are consistent with the reduction of V(V) throughout the observed particles upon magnesium intercalation during electrochemical testing. Similar trends were observed by V L_2,3_- and O K-edge X-ray absorption spectroscopy (XAS) with electron yield sensitivity, which averages over a larger footprint of the electrode than EELS. While the observation supports that the reduction of V(V) is extensive, the changes were less pronounced in our XAS than EELS measurements, suggesting the existence of heterogeneity in the extent of reaction. Reversibility was observed in both EELS and XAS, although it was more pronounced in the latter case. The extent to which heterogeneity exists and how it correlates with reversibility is the subject of follow-up work.

**Figure 5:**
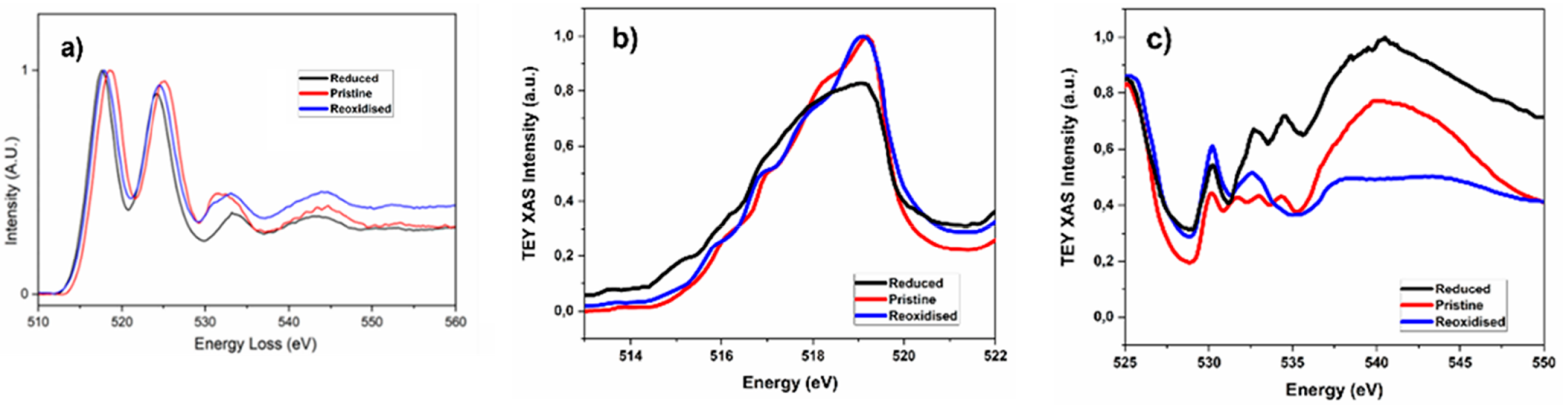
EEL spectra (a) and XAS V L- and O K-edges between 513–522 eV (b) and 525–550 eV (c) of pristine, reduced, and reoxidized particles.

*In situ* high energy X-ray diffraction experiments were also performed. This technique averages over the entire electrode volume, complementing the different length scales of EELS and XAS. Clear changes in the crystalline structure of the compound were observed upon reduction, confirming that the reaction is widespread and not constrained to shallow regions. Figure 6 depicts selected operando X-ray diffraction patterns of β-V_2_O_5_ during magnesium insertion and deinsertion corresponding to the initial state, end of the first reduction, end of reoxidation, and end of second reduction. The broad reflection centered at 10° was ascribed to the activated carbon used as the counter electrode. Upon the first reduction, a small displacement of the (100) peak to lower angles was observed, which could be associated with the elongation in the *a* axis caused by the insertion of Mg in the interlayer space. A similar phenomenon was reported to take place in sodium cells, to a much larger extent.^17^ In addition to that, broadening of the peaks was also evident, in line with the partial delamination detected by STEM measurements. Upon oxidation, the inverse trend was observed but to a lesser extent, which agrees with the partial reversibility in the electrochemical cycling. The β-V_2_O_5_ structure was preserved after Mg deintercalation, in agreement with previous works, which have shown that the structure of β-V_2_O_5_ is retained at ambient pressure, and after the long-term cycling of lithium cells, even though at ambient pressure it is metastable with respect to that of the β-form.^19,20^ The changes upon the second reduction are more significant, in agreement with the higher capacity observed. Attempts to index the pattern obtained after the second reduction yield a monoclinic cell resulting from doubling *b* axis of β-V_2_O_5_ and a change in unique axis from *b* to *c* in addition to the aforementioned enlargement of *a* parameter. The Le Bail fit using this cell and the in operando diffraction pattern obtained at the end of the second reduction are presented in Figure 7.

**Figure 6:**
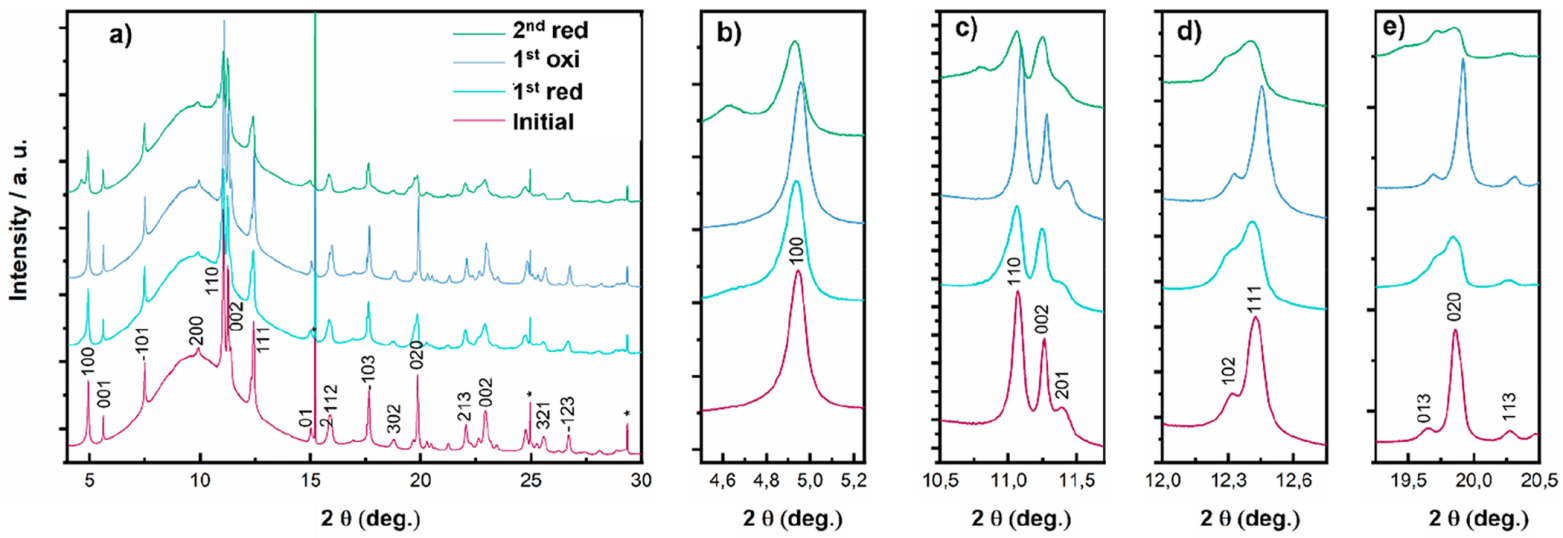
*In situ* synchrotron X-ray diffraction pattern obtained at initial state (purple), at the end of the 1st reduction (cyan), at the end of reoxidation (blue), and at the end of the 2nd reduction (green). (a) A larger 2θ range with (b–e) zoomed images of selected regions. * = Al current collector. λ = 0.6192 Å.

**Figure 7:**
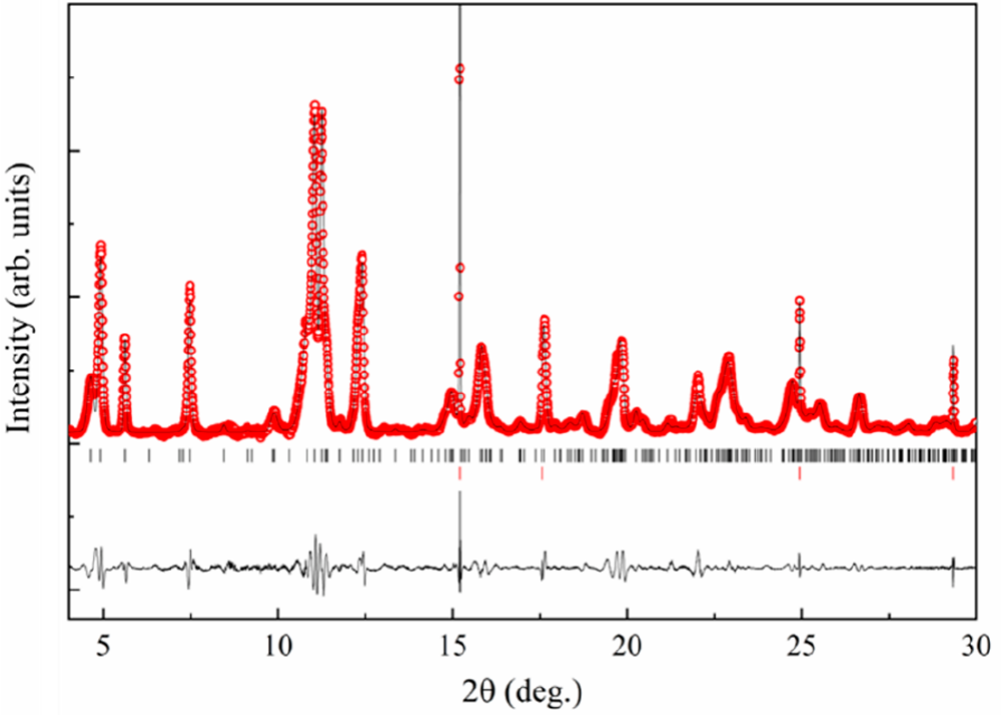
Le Bail fit of the pattern obtained after the second reduction using *a* = 7.267 Å, *b* = 7.733 Å, *c* = 6.315 Å, γ = 97.5°. Background has been subtracted to enhance the signal. Red ticks indicate the position of Al peaks coming from the current collector. λ = 0.6192 Å.

In summary, our investigations confirm that electrochemical reduction and reoxidation of β-V_2_O_5_ at room temperature induces reversible Mg^2+^ intercalation and deintercalation. While the previously reported capacities for magnesium insertion in V_2_O_5_ polymorphs using dry electrolytes at room temperature are in general low, with an average value equal to 59 mAh g^–1^,^22^ equivalent to 0.2 magnesium ions per formula unit, we found that β-V_2_O_5_ exhibits a significantly larger capacity (almost four times). The results reported herein open a path for further optimization by following strategies that successfully advanced the Li-ion technology.^23^ In this sense, β-V_2_O_5_ is a worthwhile candidate for further consideration.

## Supporting Information Available

Description of the experimental methods (PDF)
