# Supplementary material for: β-V2O5 as Magnesium Intercalation Cathode
Source: ACS Appl Energy Mater. 2022 Oct 3;5(10):11964–9. doi: 10.1021/acsaem.2c02371 (PMC9597546; doi:10.1021/acsaem.2c02371)
Supplement: Supplementary file 1 — ae2c02371_si_001.pdf [file ae2c02371_si_001.pdf]

Supplementary Information

β-V_2O_5 as Magnesium Intercalation Cathode

Rafael Trócoli[a,b], Prakash Parajuli[d], Carlos Frontera[a], Ashley P. Black[a], Grant C.B. Alexander[c,f], Indrani Roy[c],

M. Elena Arroyo-de Dompablo,[e] Robert F. Klie,[d,f] Jordi Cabana[c, f], and M. Rosa Palacín[a].

a- Instituto de Ciencia de Materiales de Barcelona (ICMAB-CSIC), Campus de la UAB, 08193 Bellaterra, Catalonia, Spain

b- Departamento de Química Inorgánica e Ingeniería Química, Instituto Universitario de Nanoquímica (IUNAN), Facultad de Ciencias, Universidad de Córdoba, Campus de Rabanales, Córdoba 14071, Spain.

c- Department of Chemistry, University of Illinois at Chicago, Chicago, Illinois 60607, United States.

d- Department of Physics, University of Illinois at Chicago, Chicago, Illinois 60607, United States.

e- Departamento de Química Inorgánica, Universidad Complutense de Madrid, Madrid, 28040, Spain

f- Joint Center for Energy Storage Research, Argonne National Laboratory, Argonne, Illinois 60439, United States

Experimental methods

To obtain β-V_2O_5, commercial (Aldrich) α-V_2O_5 was subjected to 4 GPa pressure and a temperature of 800 °C in a Conac press. After the pressure and temperature were applied for 0.5 h, the vessel was quenched to RT while pressure was slowly released. For electrode preparation, β-V_2O_5 was mixed with Super P carbon as a conductive agent and polyvinylidene fluoride (PVDF, Kynar) as a binder (in ratios 7.5:2:0.5.) in a mortar using ethanol to get a slurry, which was manually ground to dryness. The powder obtained was pressed at 8 Tons obtaining thin pellets used as electrodes which were dried overnight under vacuum at 80°C. The β-V_2O_5 mass in the electrodes was ca. 2 mg. This mass was increased to ca. 7 mg for the electrodes analyzed by the different structural characterization techniques (SXRD, EEL, XAS) reaching the needed amount to perform such studies. The electrochemical tests were performed in both, two and three electrode configurations, using Swagelok cells or coin cells assembled in an Ar-filled dry box. Pt and stainless steel were employed as current collectors for the working and reference/counter electrodes, respectively, and glass fiber separator (Whatman, GE Healthcare, 420 µm thick) soaked in 0.6 ml of electrolyte solution (0.1 M $Mg(ClO_4)_2$ (Alfa Aesar) in acetonitrile dried by immersion in molecular sieve (Sigma Aldrich). Activated Carbon cloth (Kynol, ACC-509220) was used as counter and counter/reference electrode in three and two electrodes configuration, respectively. Ag wire was employed as reference electrode in three electrode configuration. The water content of the electrolyte was measured by Karl-Fisher titration and found to be < 15 ppm of H_2O. The electrochemical tests were performed on Bio-Logic VMP3 potentiostat/galvanostats, applying potential steps of 0.1 mV s^{-1} or a constant current equivalent to C/25 for cyclic voltammetry and galvanostatic cycling measurements, being 1C equivalent to 1 mole of Mg^{2+} inserted in 1 hour (equivalent to 2 moles of electrons) *Operando* electrochemical tests were performed using 2032 coin cells with a 4-5 mm glass window. [19,20] An aluminum foil (3µm, Goodfellow) was placed on the positive electrode side to protect the window and ensure good electric contact. Same separator, counter/reference electrode, electrolyte, and cycling conditions as for the above-described standard coin cells were used. *Operando* measurements were conducted at ALBA synchrotron on the powder diffraction station of the MSPD beamline (ALBA synchrotron, Cerdayola del Vallès, Spain) [17] using the position-sensitive detector MYTHEN and $\lambda = 0.6192$ with patterns being collected in the $\approx 1.5 \leq 2\theta \leq 59°$ range in 0.006° steps and an integration time of 172 s. Scanning electron micrographs were acquired using a FEI Quanta 200 FEG microscope under a high vacuum operating at 20 kV. Scanning transmission electron microscopy (STEM) was performed using the JEOL ARM200CF, an aberration-corrected, cold-field emission instrument operated at 200 kV primary energy. Imaging was performed using a semi-convergence angle of 28 mrad. Energy-dispersive X-ray spectroscopy (EDS) was performed using an Oxford XMAX100TLE with a silicon drift detector. Electron energy-loss spectroscopy (EELS) was performed using a post-column Gatan Continuum Spectrum Imaging detector with an acceptance angle of 65 mrad. X-ray absorption spectroscopy (XAS) measurements at the V $L_{2,3}$- and O K-edge were performed at beamline 7-ID of the National Synchrotron Light Source II (NSLS-II) at Brookhaven National Laboratory (BNL) using a total electron yield (TEY) detector. Due to the proximity of the electron binding energies, O K-edges were scanned in conjunction with V L-edges.
